# New Approaches to Clarify Antinociceptive and Anti-Inflammatory Effects of the Ethanol Extract from *Vernonia condensata* Leaves

**DOI:** 10.3390/ijms12128993

**Published:** 2011-12-07

**Authors:** Jucélia Barbosa da Silva, Vanessa dos Santos Temponi, Felipe Valente Fernandes, Geórgia de Assis Dias Alves, Dalyara Mendonça de Matos, Carolina Miranda Gasparetto, Antônia Ribeiro, José de Jesus R. G. de Pinho, Maria Silvana Alves, Orlando Vieira de Sousa

**Affiliations:** 1 Department of Pharmaceutical Sciences, Faculty of Pharmacy, Federal University of Juiz de Fora, Campus Universitário, Juiz de Fora, Minas Gerais 36036–330, Brazil; E-Mails: juceliabs@yahoo.com.br (J.B.S.); vanessatemponi@hotmail.com (V.S.T.); felipevf_mg@hotmail.com (F.V.F.); georgia.assis@gmail.com (G.A.D.A.); dalyaramatos@yahoo.com.br (D.M.M.); carolina.gasparetto@ufjf.edu.br (C.M.G.); jose.pinho@ufjf.edu.br (J.J.R.G.P.); alves_ms2005@yahoo.com.br (M.S.A.); 2 Department of Biochemistry, Institute of Biological Sciences, Federal University of Juiz de Fora, Campus Universitário, Juiz de Fora, Minas Gerais 36036–330, Brazil; E-Mail: antonia.ribeiro@ufjf.edu.br

**Keywords:** *Vernonia condensata*, antinociceptive activity, sedative effect, anti-inflammatory activity

## Abstract

The present study was aimed at evaluating the antinociceptive and anti-inflammatory effects of the ethanol extract from *Vernonia condensata* leaves in animal models, in order to afford a better understanding of these properties. The extract reduced the number of abdominal contortions at doses of 100 (51.00 ± 3.00), 200 (42.00 ± 2.98) and 400 mg/kg (39.00 ± 4.00). In formalin tests, a significant reduction in the licking time (*p* < 0.01) was observed in the first phase by 25.14 (200 mg/kg = 51.50 ± 4.44) and 31.15% (400 mg/kg = 48.00 ± 4.37). The doses of 100 (43.37 ± 5.15), 200 (34.62 ± 4.16) and 400 mg/kg (28.37 ± 3.98) inhibited (*p* < 0.001) the second phase. After 60 and 90 min of treatment, a dose of 400 mg/kg (10.13 ± 0.39 and 11.14 ± 1.33, respectively) increased the latency time. Doses of 200 and 400 mg/kg potentiated the sleeping time induced by diazepam, pentobarbital and meprobamate. The extracts (100, 200 and 400 mg/kg) showed anti-inflammatory effects by a decrease in paw edema. The extracts also reduced the exudate volume at the doses of 200 and 400 mg/kg. The leukocyte migration had significant effect (*p* < 0.001) at doses of 100, 200 and 400 mg/kg. The completion of additional experiments in the investigation of the antinociceptive and anti-inflammatory activities of *V. condensata* allowed a better understanding of the central and peripheral mechanisms involved.

## 1. Introduction

Pain is considered to be one of the most important symptoms associated with inflammatory diseases [1,2] and affects a large portion of the population, diminishing their quality of life [3,4]. The use of anti-inflammatory drugs has been required to inhibit the mediators of the inflammation, preventing the acute response and the development of the chronic process [5]. Therefore, based on traditional medicine, search for new natural products from medicinal plants with analgesic and anti-inflammatory properties have been encouraged [6–8].

Plants of the genus *Vernonia* (Asteraceae), having approximately 1000 species, are widely distributed in most tropical and subtropical countries, and have long been popularly used to treat several types of diseases [9]. *Vernonia* has been used to treat a number of disorders including inflammation, malaria, fever, worms, pain, diuresis, cancer, abortion, and various gastro-intestinal problems [10,11]. In addition, species of this genus have been studied and their pharmacological properties established. For example, *V. glabra* showed hypotensive effect [12], *V. patens* demonstrated phototoxic, antibacterial and anti-inflammatory activities [13] and *V. kotschyana* presented immunomodulatory activity [14]. *V. amygdalina* revealed anti-histaminic effect [15] but was not hepatotoxic in rats [16]. These activities can be attributed to compounds such as polysaccharides from *V. kotschyana* [14], vernolepin found in *V. amygdalina* [15] and hesperidin, 3′-methylhesperetin, homoesperetin-7-*O*-rutinoside, sitosterol and stigmasterol identified in *V. diffusa* [17].

*Vernonia condensata* Baker, one Asteraceae family member, commonly known as figatil or necroton, has been traditionally used as analgesic, anti-inflammatory, anti-thermal, antianemics, antibacterial, liver tonic, liver toxicity and antiulcerogenic agents [10,11]. Analgesic, anti-inflammatory and antiulcerogenic activities of *V. condensata*, as well as toxicity have been investigated [18–21]. In these studies, vernonioside B2 demonstrated antinociceptive and anti-inflammatory effect [20]. Furthermore, constituents such as saponins, tannins, alkaloids, phenolic compounds and flavonoids were detected in the extracts from *V. condensata* [21].

Although antinociceptive and anti-inflammatory activities from *V. condensata* have been described, new essential investigations have been encouraged in order to confirm these evidences and to establish the involved mechanisms by methods not yet applied. In this sense, in the present study we investigated the antinociceptive, anti-inflammatory and sedative properties of the ethanol extract from *V. condensata* leaves, using appropriate experimental animal models.

## 2. Results and Discussion

### 2.1. Phytochemistry Screening

The phytochemical screening results of the ethanol extract from *V. condensata* leaves showed the presence of different types of active constituents such as flavonoids, terpenoids, sterols, coumarins, tannins, saponins and volatile oils.

### 2.2. Acute Toxicity

At the doses administered per oral route (p.o.), the ethanol extract from *V. condensata* leaves was not toxic to animals, presenting LD_50_ up to 3 g/kg. During this experiment, an important feature was observed that the mice presented somnolence. The dosage definition in the experiments of pharmacological activities was based on the LD_50_ value.

### 2.3. Writhing Response Induced by Acetic Acid in Mice

**Figure 1:**
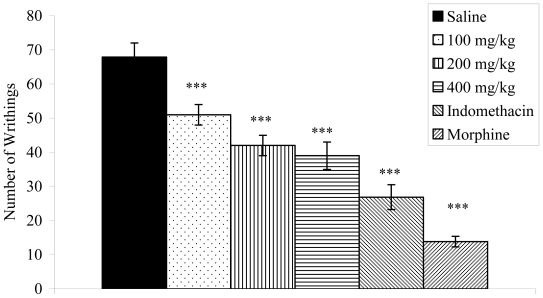
Effects of the ethanol extract from *V. condensata* leaves on acetic acid induced writhing in mice. Data are mean ± S.E.M. of eight mice. ***P < 0.001 *vs.* control group.

Doses of 100 (51.00 ± 3.46; 24.86%), 200 (42.00 ± 2.98; 38.12%) and 400 mg/kg (39.00 ± 4.00; 42.54%) of *V. condensata* ethanol extract significantly (*p* < 0.001) reduced the abdominal contortions induced by acetic acid when compared to the control group (67.87 ± 4.14) (Figure 1).

### 2.4. Effects on Formalin-Induced Nociception in Mice

**Figure 2:**
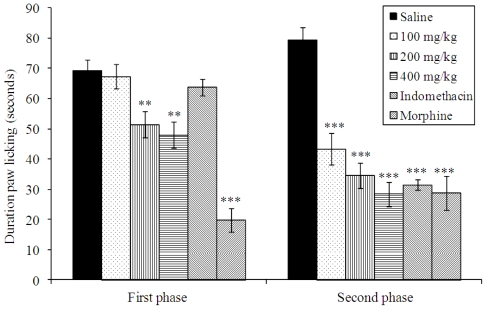
Effects of the ethanol extract from *V. condensata* leaves on formalin-induced nociception in mice. First phase = 0–5 min after formalin injection; second phase = 15–30 min. Data are mean ± S.E.M. of eight mice. **P < 0.01; ***P < 0.001 *vs.* control group.

The intraplantar injection of formalin promoted a biphasic characteristic response (Figure 2). The time spent licking in the first phase (0–5 min) was 66.62 ± 4.76 s and in the second phase (15–30 min) was 79.37 ± 4.15 s for the control group. After 60 min of treatment, a significant reduction in the licking time (*p* < 0.01) was observed during the first phase after formalin administration (neurogenic) by 25.14 and 31.15% with 200 and 400 mg/kg of extract, respectively (Figure 2). In the second phase, the doses of 100, 200 and 400 mg/kg of extract inhibited significantly (*p* < 0.001) at 45.36, 56.38 and 64.26%, respectively, when compared to the control. As expected, morphine (1 mg/kg, s.c.) significantly reduced the formalin response in both phases. The indomethacin inhibitory effect was observed in the second phase.

### 2.5. Effects on Hot-Plate Latency Assay in Mice

The *V. condensata* ethanol extract increased the latency time of mice exposed to the hot plate test (Table 1). After 60 and 90 min of treatment, dose of 400 mg/kg (82.52 and 87.54%, respectively) increased significantly (*p* < 0.001) the latency time in the respective control group. Morphine proved to be a potent analgesic, increasing the latency time within the evaluation periods. Naloxone, an opioid antagonist, blocked the morphine action but did not alter the antinociceptive effect of the tested extract, increasing the latency time at 102.52%. In this assay, we also observed that mice became lethargic.

### 2.6. Effect on Sleeping Time

**Figure 3:**
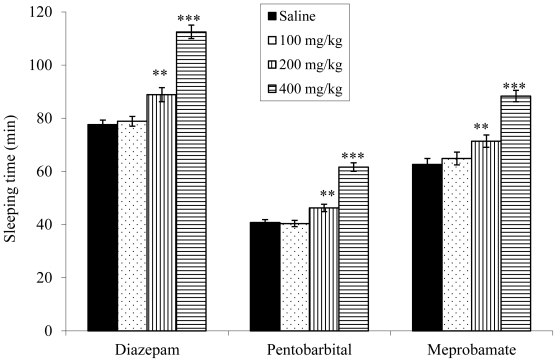
Effect of the ethanol extract from *V. condensata* leaves on sleeping time (min) induced by diazepam, pentobarbital and meprobamate in mice. Data are mean ± S.E.M. of eight mice. **P < 0.01; ***P < 0.001 *vs.* control group.

The doses of 200 and 400 mg/kg of ethanol extract from *V. condensata* potentiated the sleeping time induced by diazepam (57.7%, 98%, and 156.4%, respectively), pentobarbital (75.4%, 122.9%, and 192.6%, respectively) and meprobamate (56.1%, 89.6%, and 124.4%, respectively) (Figure 3).

### 2.7. Effects on Carrageenan-Induced Edema in Rats

The *V. condensata* ethanol extract anti-inflammatory effect evaluated by the paw edema method induced by carrageenan is shown in Table 2. Based on the presented data, the edema inhibition was observed 2 h after carrageenan application of doses of 200 (0.51 ± 0.06; 40.00%; *p* < 0.05) and 400 mg/kg (0.59 ± 0.07; 30.59%; *p* < 0.05). Three hours after carrageenan injections, the doses of 100 (0.73 ± 0.07; *p* < 0.05), 200 (0.72 ± 0.03; *p* < 0.05) and 400 mg/kg (0.63 ± 0.04; *p* < 0.01) reduced the respective paw edema (21.50, 22.58 and 32.26%). The paw edema was also inhibited at the doses 100, 200 and 400 mg/kg 4 h after carrageenan application. In this time, indomethacin reduced the paw edema at 50.00%.

### 2.8. Effects on Carrageenan-Induced Pleurisy in Rats

**Figure 4:**
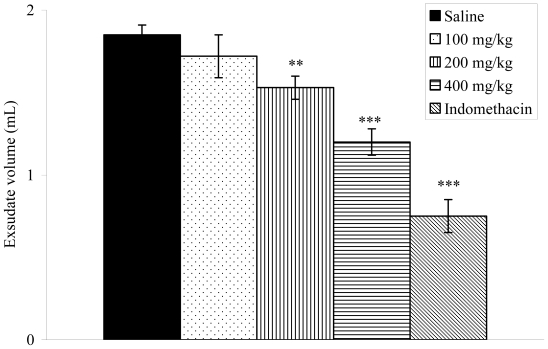
Effects of the ethanol extract from *V. condensata* leaves on pleural exudation in carrageenan-induced pleurisy in rats. Data are mean ± S.E.M. of six rats. ** *P* < 0.01, *** *P* < 0.001 *vs.* control group.

**Figure 5:**
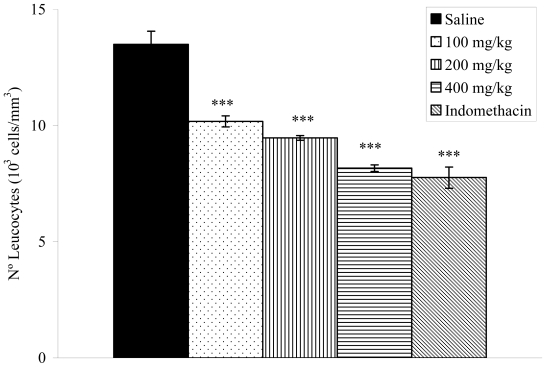
Effects of the ethanol extract from *V. condensata* leaves on number of leucocytes in carrageenan-induced pleurisy in rats. Data are mean ± S.E.M. of six rats. *** *P* < 0.001 *vs.* control group.

The anti-inflammatory effect of the *V. condensata* ethanol extract was confirmed by a decrease in exudate volume and leukocyte migration to the pleural cavity of rats. The pleurisy effects demonstrated that doses of 200 (1.53 ± 0.07; *p* < 0.01) and 400 mg/kg (1.20 ± 0.08; *p* < 0.001) of the extract significantly reduced the exudate volume by 17.30 and 35.13%, respectively, when compared to the control group (Figure 4). The number of total leukocytes was inhibited significantly (*p* < 0.001) at the doses of 100 (10.18 ± 0.24 × 10^3^ cells/mm^3^), 200 (9.47 ± 0.10 × 10^3^ cells/mm^3^) and 400 mg/kg (8.17 ± 0.14 × 10^3^ cells/mm^3^) (Figure 5). Indomethacin reduced the exudate volume and the leukocyte migration.

Considering that the use of commercially available analgesic and anti-inflammatory drugs (opioids and non-steroidal anti-inflammatory drugs) exerts a wide range of side effects [5], there is currently a strong interest in developing new therapeutic agents from natural products [6–8]. Agents that inhibit different mediators that are involved in the evolution of inflammatory processes, including pain, are especially relevant [2,5]. In this context, studies have been carried out with natural products in models of pain and inflammation in order to assess their pharmacological potential, as well as developing new therapeutic options [6–8,22,23].

The phytochemical analyses revealed the presence of flavonoids, tannins, coumarins, saponins, alkaloids, glycosides, tannins, triterpenoids and steroidal nucleus. Despite previous descriptions of most of these constituents [21], in the present study coumarins and glycosides were detected for the first time. A variety of *in vitro* and *in vivo* experiments have shown that flavonoids, tannins, triterpenoids and other secondary plant metabolites possess analgesic and anti-inflammatory properties [23–25]. Vernonioside B2, for example, identified in this plant, may contribute to the observed anti-inflammatory and antinociceptive effects of *V. condensata* [20].

The acute toxicity test showed that the *V. condensata* leaves’ ethanol extract doses determined were not toxic to mice, confirming results of earlier studies [19,21]. It is important to mention that the largest dose administered (400 mg/kg) is less than the lowest dose applied for determination of the LD_50_ (0.5 g/kg or 500 mg/kg). During the experiment, it was observed that the mice presented somnolence, demonstrating a probable sedative effect.

In this study, we investigated an ethanol extract from *V. condensata* by classical nociception and acute inflammation models. This study demonstrated that ethanol extract produces antinociceptive and anti-inflammatory effects in models of nociception (acetic acid-induced abdominal writhing, formalin test and hot plate test) and inflammation (paw edema and pleurisy tests), providing a scientific basis to explain, in part, the popular use of *V. condensata* in Brazilian folk medicine. It also suggests that the ethanol extract contains bioactive constituents that could be responsible for the observed activities.

The acetic acid-induced writhing reaction has been largely used as a screening tool for the assessment of analgesic or anti-inflammatory properties. According to Collier *et al.* [26], acetic acid acts indirectly by inducing the release of endogenous mediators sensitive to nonsteroidal anti-inflammatory drugs and opioids. This substance also promotes an increase in exudates levels of PGE_2_ and PGF_2_ (mediators of inflammation), bradykinin, substance P and some cytokines (IL-1β, TNF-α, and IL-8) [27]. For this experiment, ethanol extract from *V. condensata* pretreated animals modified the nociceptive response induced by acetic acid in a dose-dependent manner. This fact suggests that probably the antinociceptive action of extract occurs peripherally, and it also inhibits the release of mediators in response to acetic acid. Therefore, the result corroborates previously studies [18,20,21].

Formalin-induced nociception was another essential test employed in this study and this classical animal model was not applied in the reports made before with *V. condensata*. It is considered the most predictive of acute pain because it causes a local tissue injury to the paw and is also indicative of tonic and localized inflammation pain [28]. This test exhibits two distinct phases of the licking response: the first phase is characterized by neurogenic pain caused by a direct chemical stimulation of nociceptors, and the second phase is characterized by inflammatory pain generated by a combination of stimuli, including inflammation of the peripheral tissues and mechanisms of central sensitization. In this last phase, different chemical mediators are involved, such as excitatory amino acids, neuropeptides, PGE2, nitric oxide, and kinins [29]. We observed that when ethanol extract from *V. condensata* was injected 60 min prior to formalin, a significant inhibition of the formalin response was seen during the second phase (inflammatory). It is suggested that the early phase is due to a direct effect on nociceptors and prostaglandins that do not play an important role during this phase. In contrast, the late phase seems to be an inflammatory response that can be inhibited by nonsteroidal anti-inflammatory drugs and corticosteroids [28,29]. Taken together, these results revealed a probably similar action to the nonsteroidal anti-inflammatory drugs.

To distinguish between central and peripheral antinociceptive actions, we extended our studies to the hot-plate test because this technique has not been used in previous studies with *V. condensata*. This test is considered to be sensitive to drugs acting at the supraspinal modulation level of the pain response [27], suggesting at least a modulatory effect of the extract. In this study, antinociceptive action did not depend entirely on the opioid system, because the treatment with naloxone did not completely reverse the produced effect [6]. Furthermore, after thermal stimulus, mice showed lethargy, indicating a sedative effect, which could explain the analgesic activity independent of opioid action.

To better understand the somnolence and lethargy by observing signals in the experiments of acute toxicity and hot plate test, and to clarify the analgesic activity independent of opioid system activity, diazepam, pentobarbital and meprobamate were used to induce sleep in the animals. Benzodiazepines act at specific binding sites that are closely linked to γ-aminobutyric acid (GABA_A_) receptor and enhancing the GABA-ergic transmission which might be related to its sedative activity [30]. Prolongation of pentobarbital-induced sleeping time might be due to the tranquilizing action as well as CNS depressant action related to GABA_A_ receptors [31]. Although the exact mechanism responsible for the sedation action of meprobamate is not fully clear, it might be due to CNS depressant action or also due to enhancement of GABA-ergic transmission [32]. The ethanol extract from *V. condensata* significantly potentiated the duration of diazepam-, pentobarbital- and meprobamate-induced sleep in mice, suggesting probable tranquilizing action as well as CNS depressant action [30–32]. However, the responsible compound(s) for the hypnotic effect of *V. condensata* is not clearly known and could not be concluded based on the results of the present study. On the other hand, other plants containing compounds such as flavonoids, terpenes and saponins have been found to have hypnotic effects [33]. Therefore, we can speculate that these constituents might be responsible for the sedative effect of *V. condensata* since these chemical classes were detected in the present study. Flavonoids with anxiolytic and/or antidepressant activities have also been described in numerous plant species used in folk medicine to depress the CNS. This effect has been described by their affinity for the central benzodiazepine receptors [34]. It could be suggested that flavonoids of the *V. condensata* contribute to the sedative effect of this plant through benzodiazepine receptors.

The anti-inflammatory effect observed in the formalin test was confirmed in carrageenan-induced paw edema in rats, an animal model widely employed for the screening of anti-inflammatory compounds. The inflammatory response induced by carrageenan is characterized by the formation of marked edema resulting from the release of several mediators such as histamine, serotonin and bradykinin; this is subsequently sustained by release of prostaglandins produced by inducible isoforms of cyclooxygenase (COX-2) [35–37]. In the present study, oral treatment with the ethanol extract from *V. condensata* inhibited carrageenan-induced paw edema in rats. This treatment consistently attenuated the paw edema induced by carrageenan, as well as by several inflammatory mediators known to participate in the carrageenan response, such as bradykinin, histamine, substance P and platelet-activating factor [38–41]. This evidence suggests that the anti-inflammatory actions of the ethanol extract from *V. condensata* are related to the inhibition of one or more intracellular signaling pathways involved in the effects of these mediators.

The inflammation model of carrageenan-induced pleurisy was used to gain further insights into the anti-inflammatory effects of the ethanol extract from *V. condensata* [42–44]. Carrageenan-induced pleurisy has been used to investigate the mechanisms involved in acute inflammation and also to assess the effectiveness of anti-inflammatory drugs [42,45]. As expected, in our experiments intrapleural injection of carrageenan caused a marked accumulation of pleural exudate, followed by intense migration of inflammatory cells into the pleural cavity. Treatment of rats with the ethanol extract significantly reduced the volume of pleural exudate accumulated in response to carrageenan injection and also inhibited the migration of leucocytes.

## 3. Experimental Section

### 3.1. Plant Material and Extraction

Specimens of *Vernonia condensata* Baker used in this study were cultivated and collected at the Medicinal Garden of the Faculty of Pharmacy, Federal University of Juiz de Fora, in Juiz de Fora, State of Minas Gerais, Brazil, in August 2010. The species was identified by Dr. Fátima Regina Gonçalves Salimena and a voucher specimen (CESJ number 52943) was deposited in the Herbarium of the Federal University of Juiz de Fora, Brazil. Dried and powdered mature leaves (465 g) were exhaustively extracted in 95% ethanol (2.5 L) by static maceration for 3 weeks at room temperature with renewal of solvent every 2 days. The ethanol extract was filtered and evaporated under a rotary evaporator at controlled temperature (50–60 °C). This material was placed in a desiccator with silica to yield 27 g. The dried extract was dissolved using 1% DMSO in normal saline for pharmacological studies.

### 3.2. Chemicals

Drugs and reagents used in this study (and their sources) were as follows: acetic acid (Vetec Química Farm Ltda, Rio de Janeiro, RJ, Brazil), formaldehyde (Reagen Quimibrás Ind. Química S.A., Rio de Janeiro, RJ, Brazil), diazepam (União Química Farmacêutica Nacional S/A, Embu-Guaçu, SP, Brazil), pentobarbital (Syntec, Cotia, SP, Brazil), morphine hydrochloride (Merck Inc., Whitehouse Station, NJ, USA), meprobamate, naloxone and indomethacin (Sigma Chemical Co, St Louis, MI, USA).

### 3.3. Phytochemical Screening of the Ethanol Extract

The screening of chemical constituents was carried out with the ethanol extracts using chemical methods and thin-layer chromatography (TLC), according to the methodology suggested by Matos [46], including flavonoids, tannins, coumarins, alkaloids, saponins, terpenoids, steroids and volatile oils.

### 3.4. Animals

Male Wistar rats (90–110 days) weighing 200–240 g and male Swiss albino mice (50–70 days) weighing 25–30 g were used in the experiments. The animals were provided by the Central Biotery of the Federal University of Juiz de Fora. The animals were divided into groups and kept in plastic under a 12 h light/12 h dark cycle at room temperature (22 ± 2 °C), with free cages (47 × 34 × 18 cm) access to Purina® rations and water. Animal care and the experimental protocol followed the principles and international guidelines suggested by the Brazilian College of Animal Experimentation (COBEA) and were approved by the local ethical committee (protocol number 036/2010).

### 3.5. Acute Toxicity

Groups of ten mice received oral doses of 0.5, 1, 1.5, 2 and 3 g/kg of ethanol extract from *V. condensata*, while the control group received the vehicle (saline). The groups were observed for 48 h and 50% lethal dose (LD_50_) was mortality at end of this period was recorded for each group [47]. The LD_50_ determined by probit test using a log plot of percentage death *versus* dose [48].

### 3.6. Acetic Acid-Induced Writhing Test

The acetic-acid writhing test is used for the evaluation of analgesic activity [26]. Mice (n = 8 per group) were injected (i.p.) with 0.6% acetic acid (10 mL/kg body weight), and the intensity of nociception was quantified by counting the total number of writhes that occurred between 10 and 30 min after injection. Animals received ethanol extract (100, 200 or 400 mg/kg, p.o.) or sterile saline (control group, 0.9%, w/v) 60 min before acetic acid injection. Indomethacin (10 mg/kg, p.o.) and morphine (1 mg/kg, s.c.) were administered 60 min before acetic acid as reference compounds.

### 3.7. Formalin Test

Twenty microliters of 1% formalin was administered i.pl. in the mouse’s right paw. The licking time was then recorded from 0 to 5 min (phase 1, neurogenic) and from 20 to 25 min (phase 2, inflammatory) after formalin administration [29,49]. Mice were then treated (p.o.) with extract (100, 200 or 400 mg/kg) or sterile saline (0.9%) 60 min before formalin injection. Indomethacin (10 mg/kg, p.o.) and morphine (1 mg/kg, s.c.) were also administered 60 min before the formalin injection and used as reference compounds.

### 3.8. Hot Plate Test

Animals were placed on a hot-plate (Model LE 7406, Letica Scientific Instruments, Barcelona, Spain) heated at 55 ± 1 °C [50]. Three groups of mice (n = 8) were treated p.o. with ethanol extract (100, 200 or 400 mg/kg; 0.1 mL per 10 g body weight); the control group received sterile saline (10 mL/kg). Measurements were performed at time 0, 30, 60 and 90 min after drug administration, with a cut-off time of 40 s to avoid lesions to the animals’ paws. The effect of pretreatment with naloxone (1 mg/kg, subcutaneously) on the analgesia produced by the ethanol extract (400 mg/kg) was determined in a separate group of animals. Morphine (1 mg/kg, subcutaneously), in the absence and presence of naloxone treatment, was used as a reference.

### 3.9. Effect on Sleeping Time in Mice

Mice were divided into 4 groups, each group containing 8 animals. The animals of group I served as the control (normal saline, 0.9 % (w/v) NaCl, 5 mL/kg); groups II, III, and IV received ethanol extract at the doses (100 mg/kg, 200 mg/kg and 400 mg/kg, respectively). Normal saline and the extracts were applied orally 60 min prior to the administration of pentobarbital sodium (40 mg/kg, i.p.), diazepam (3 mg/kg, i.p.) and meprobamate (100 mg/kg, i.p.). The sleeping time was noted by recording the interval between the losses and regaining of righting reflex [51].

### 3.10. Carrageenan-Induced Rat Paw Edema

Anti-inflammatory activity was assessed on the basis of inhibition of paw edema induced by the injection of 0.1 mL of 2% carrageenan (an edematogenic agent) into the subplantar region of the right hind paw of the rat [52]. Male Wistar rats were divided into groups of six animals which received p.o. doses of extract (100, 200 and 400 mg/kg; 0.1 mL per 10 g body weight), saline or indomethacin (10 mg/kg) 1 h before the injection of carrageenan. In the left paw, used as a control, 0.1 mL of sterile saline was injected. 1, 2, 3 and 4 h after injection of carrageenan, the measure of edema was made by the difference between the volume displaced by the right paw and the left paw using a plethysmometer (model LE 7500, Letica Scientific Instruments, Barcelona, Spain).

### 3.11. Carrageenan-Induced Pleurisy in Rats

Pleurisy was induced in male Wistar rats by intrapleural administration of 0.5 mL 2% carrageenan suspension in saline solution between the third and fifth ribs on the right side of the mediastinum [42]. Extract (100, 200 and 400 mg/kg), saline or indomethacin (10 mg/kg) p.o. were given 60 min before injection of the irritant. Animals were killed 4 h after carrageenan injection, and the skin and pectoral muscles were retracted. A longitudinal incision was made between the third and fifth ribs on each side of the mediastinum. The exudate was collected and transferred to a 15 mL conical centrifuge tube and the total volume determined. A 20 μL aliquot of the exudate was used to determine the total leucocyte count in Neubauer chambers.

### 3.12. Calculus and Statistical Analysis

Data are expressed as mean ± S.E.M. Statistical significance was determined by one-way analysis of variance followed by the Student–Newman–Keuls test. *P* values below 0.05 were considered significant. The percentage of inhibition was calculated by using

100-T×100/C(%) or T×100/C-100(%)

where C and T indicate non-treated (vehicle) and drug-treated, respectively.

## 4. Conclusions

The present study demonstrated that the completion of additional experiments in the investigation of the antinociceptive and anti-inflammatory activities of *V. condensata* allowed a better understanding of the central and peripheral mechanisms involved. The results support the popular use of this plant, but phytochemical studies together with pharmacological and toxicological investigations have proven essential for the complete understanding of their medicinal application.

## Figures and Tables

**Table 1:** Effects of the ethanol extract from *V. condensata* leaves on the latency time of mice exposed to the hot plate test.

Group	Dose (mg/kg)	Time after drug administration (seconds)			
		0 min	30 min	60 min	90 min
Control	Saline	5.01 ± 0.31	5.56 ± 0.83	5.55 ± 0.47	5.94 ± 0.64
	100	5.03 ± 0.46	4.88 ± 0.84	5.71 ± 0.40	5.94 ± 0.87
Ethanol extract	200	5.46 ± 0.54	6.01 ± 0.38	6.85 ± 0.45	7.50 ± 0.77
	400	5.50 ± 0.67	7.64 ± 0.99	10.13 ± 0.39***	11.14 ± 1.33***
Morphine	1	6.24 ± 0.77	9.77 ± 0.44***	11.82 ± 0.39***	14.51 ± 0.94***
Naloxone + Morphine	1 + 1	5.57 ± 0.68	9.99 ± 0.96***	8.24 ± 0.56**	7.01 ± 0.59
Naloxone + Extract	1 + 400	5.54 ± 0.74	8.39 ± 0.67*	12.16 ± 0.74***	12.03 ± 1.05***

Each value represents the mean ± S.E.M. of 8 mice.

* *P* < 0.05;

** *P* < 0.01;

*** *P* < 0.001 significantly different from the control group.

**Table 2:** Effects of the ethanol extract from *V. condensata* leaves on the rat paw edema induced by carrageenan.

Group	Dose (mg/kg)	Volume of hind paw (mL)			
		1 h	2 h	3 h	4 h
Control	Saline	0.65 ± 0.09	0.85 ± 0.09	0.93 ± 0.06	1.10 ± 0.08
	100	0.42 ± 0.02	0.72 ± 0.03	0.73 ± 0.07 *	0.85 ± 0.05 *
Ethanol extract	200	0.46 ± 0.04	0.51 ± 0.06 *	0.72 ± 0.03 *	0.70 ± 0.09 **
	400	0.45 ± 0.03	0.59 ± 0.07 *	0.63 ± 0.04 **	0.74 ± 0.06 **
Indomethacin	10	0.41 ± 0.09	0.43 ± 0.08 **	0.51 ± 0.08 ***	0.55 ± 0.06 ***

Each value represents the mean ± S.E.M. of 6 rats.

* *P* < 0.05;

** *P* < 0.01;

*** *P* < 0.001 significantly different from the control group.
